# Low 2018/19 vaccine effectiveness against influenza A(H3N2) among 15–64-year-olds in Europe: exploration by birth cohort

**DOI:** 10.2807/1560-7917.ES.2019.24.48.1900604

**Published:** 2019-11-28

**Authors:** Esther Kissling, Francisco Pozo, Silke Buda, Ana-Maria Vilcu, Alin Gherasim, Mia Brytting, Lisa Domegan, Verónica Gómez, Adam Meijer, Mihaela Lazar, Vesna Višekruna Vučina, Ralf Dürrwald, Sylvie van der Werf, Amparo Larrauri, Theresa Enkirch, Joan O’Donnell, Raquel Guiomar, Mariëtte Hooiveld, Goranka Petrović, Elena Stoian, Pasi Penttinen, Marta Valenciano, I-MOVE primary care study team

**Affiliations:** 1 Epidemiology Department, Epiconcept, Paris, France; 2 National Centre for Microbiology, National Influenza Reference Laboratory, WHO-National Influenza Centre, Institute of Health Carlos III, Madrid, Spain; 3 Robert Koch Institute, Department of Infectious Disease Epidemiology, Respiratory Infections Unit, Berlin, Germany; 4 Sorbonne Université, INSERM, Institut Pierre Louis d’épidémiologie et de Santé Publique (IPLESP UMRS 1136), Paris, France; 5 National Epidemiology Centre, Institute of Health Carlos III, Madrid, Spain; 6 CIBER de Epidemiología y Salud Pública (CIBERESP), Institute of Health Carlos III, Madrid, Spain; 7 Public Health Agency of Sweden, Stockholm, Sweden; 8 Health Service Executive- Health Protection Surveillance Centre, Dublin, Ireland; 9 European Programme for Intervention Epidemiology Training (EPIET), European Centre for Disease Prevention and Control (ECDC), Stockholm, Sweden; 10 Departamento de Epidemiologia, Instituto Nacional de Saúde Dr. Ricardo Jorge, Lisbon, Portugal; 11 National Institute for Public Health and the Environment (RIVM), Bilthoven, the Netherlands; 12 ”Cantacuzino” National Military-Medical Institute for Research and Development, Bucharest, Romania; 13 Croatian Institute of Public Health, Division for epidemiology of communicable diseases, Zagreb, Croatia; 14 Robert Koch Institute, National Reference Center for Influenza, Germany; 15 Unité de Génétique Moléculaire des Virus à ARN, Institut Pasteur, CNRS UMR3569, Université Paris Diderot SPC, France; 16 CNR des virus des infections respiratoires, WHO National Influenza Center, Institut Pasteur, Paris, France; 17 Departamento de Doenças Infeciosas, Instituto Nacional de Saúde Dr. Ricardo Jorge, Lisbon, Portugal; 18 Nivel (Netherlands Institute for Health Services Research), Utrecht, the Netherlands; 19 European Centre for Disease Prevention and Control (ECDC), Stockholm, Sweden; 20 The I-MOVE primary care study team members are listed at the end of the article

**Keywords:** influenza, vaccine effectiveness, birth cohorts, imprinting, A(H3N2), multicentre study

## Abstract

**Introduction:**

Influenza A(H3N2) clades 3C.2a and 3C.3a co-circulated in Europe in 2018/19. Immunological imprinting by first childhood influenza infection may induce future birth cohort differences in vaccine effectiveness (VE).

**Aim:**

The I-MOVE multicentre primary care test-negative study assessed 2018/19 influenza A(H3N2) VE by age and genetic subgroups to explore VE by birth cohort.

**Methods:**

We measured VE against influenza A(H3N2) and (sub)clades. We stratified VE by usual age groups (0–14, 15–64, ≥ 65-years). To assess the imprint-regulated effect of vaccine (I-REV) hypothesis, we further stratified the middle-aged group, notably including 32–54-year-olds (1964–86) sharing potential childhood imprinting to serine at haemagglutinin position 159.

**Results:**

Influenza A(H3N2) VE among all ages was −1% (95% confidence interval (CI): −24 to 18) and 46% (95% CI: 8–68), −26% (95% CI: −66 to 4) and 20% (95% CI: −20 to 46) among 0–14, 15–64 and ≥ 65-year-olds, respectively. Among 15–64-year-olds, VE against clades 3C.2a1b and 3C.3a was 15% (95% CI: −34 to 50) and −74% (95% CI: −259 to 16), respectively. VE was −18% (95% CI: −140 to 41), −53% (95% CI: −131 to −2) and −12% (95% CI: −74 to 28) among 15–31-year-olds (1987–2003), 32–54-year-olds (1964–86) and 55–64-year-olds (1954–63), respectively.

**Discussion:**

The lowest 2018/19 influenza A(H3N2) VE was against clade 3C.3a and among those born 1964–86, corresponding to the I-REV hypothesis. The low influenza A(H3N2) VE in 15–64-year-olds and the public health impact of the I-REV hypothesis warrant further study.

## Introduction

The 2018/19 influenza season in Europe was characterised by both A(H1N1)pdm09 and A(H3N2) virus subtypes circulating, with co-circulation in some countries and dominance of either A(H1N1)pdm09 or A(H3N2) influenza in other countries [1]. Few influenza B viruses were detected in Europe. Influenza A(H3N2) viruses in subclades of clade 3C.2a were circulating in Europe, but also 3C.3a clade viruses that are antigenically distinct [1,2]. The World Health Organization recommended an A/Singapore/INFIMH-16–0019/2016 (H3N2)-like clade 3C.2a virus as the A(H3N2) vaccine component for the 2018/19 northern hemisphere season [3].

Since 2008/09, the Influenza Monitoring Vaccine Effectiveness in Europe (I-MOVE) primary care multicentre case control study (MCCS) has provided vaccine effectiveness (VE) estimates by influenza virus (sub)type, age group and target population. Since 2015/16, I-MOVE has also estimated VE by virus genetic clade [4,5].

The interim VE estimate against influenza A(H3N2) up to week 4/2019 from the European multicentre I-MOVE study was −3% (95% confidence interval (CI): −100 to 47) [6]. The I-MOVE end-of-season VE estimates against influenza A(H3N2) indicated a much lower VE among 15–64-year-olds than among flanking age groups. In April 2019, we contacted other northern hemisphere study sites measuring VE to share our findings and to query if they observed similar results. The same pattern was seen among end-of-season age-specific VE estimates against influenza A(H3N2) in Canada and the United States (US) [7,29].

During summer 2019, Skowronski et al. in Canada investigated an underlying birth cohort effect, potentially related to childhood imprinting, to explain the low VE among the adult age group, as articulated in their recent publication [7]. Imprinting is the effect of antigens of an individual’s first influenza infection shaping immune memory, which is retained over the individual’s lifetime. Given the often sequential emergence and re-emergence of different influenza A subtypes and antigenic subclusters across history, there can be much variability in imprinting between birth cohorts [9]. Imprinting may influence immune- and clinical responses to subsequent influenza infections; it has been discussed since the 1950s and has been widely reported on in recent years [10-16]. Skowronski et al.’s hypothesis of imprint-regulated effect of vaccine (I-REV) specifically invokes childhood imprinting to a serine (S) residue at position 159 of antigenic site B in the haemagglutinin (HA) of circulating influenza A(H3N2) viruses and to a lesser extent also potential imprinting to S at position 193 also within HA antigenic site B. The hypothesis assumes that the S159-specific immune response resulting from this childhood imprinting protected unvaccinated adults against circulating S159-bearing 3C.3a viruses during the 2018/19 influenza A(H3N2) epidemic but that the 2018/19 Y159-mismatched vaccine interfered with that pre-immunity. The Canadian research team observed a 4.46-fold increased risk of 3C.3a illness (95% CI: 1.58 to 13.21) among vaccinated compared with unvaccinated 35–54-year-olds, roughly corresponding in 2018/19 to the birth cohort 1964–83 [7].

Since the influenza A(H3N2) pandemic in 1968, when influenza A(H3N2) first emerged, several amino acid substitutions occurred at position 159 in antigenic site B in the HA of A(H3N2) viruses circulating in Europe [17], changing from a predominance of S to tyrosine (Y) in 1987 and then to phenylalanine (F) in 2004 until and including the 2013/14 season. Since 2014/15, the predominant amino acid at position 159 in antigenic site B in the HA among influenza A(H3N2) viruses circulating in Europe has been Y among 3C.2a viruses and S among 3C.3a viruses. Amino acid change from S to Y at this position in 1987 has been identified as the cause of cluster transition owing to a large impact on the antigenic characteristics [18,19], although other amino acid changes of the HA might have played a role in the net effect of S159Y [20]. As well as harbouring the F159S substitution, 3C.3a viruses circulating in 2018/19 season harboured the F193S substitution, a position substituted several times since 1968; the F193S substitution is included in the I-REV hypothesis.

Using the I-MOVE MCCS, we report 2018/19 end-of-season VE against influenza A(H3N2), overall, by age group and for A(H3N2) (sub)clades. We further test the I-REV hypothesis that the birth cohort first exposed to A(H3N2) viruses with S at position 159 in the HA antigenic site B experienced the lowest VE against influenza A(H3N2) in this season [7].

## Methods

The I-MOVE methods have been discussed in detail elsewhere [21,22]. Briefly, study sites in nine European countries took part in the primary care-based I-MOVE multicentre study in the 2018/19 influenza season: Croatia, France, Germany, Ireland, the Netherlands, Portugal, Romania, Spain and Sweden. General practitioners (GPs) or paediatricians systematically selected patients with influenza-like illness (ILI) or acute respiratory infection (ARI) to interview and swab. Using the test-negative design, patients positive for influenza virus were classified as cases (by influenza virus (sub)type), those negative as controls [23].

### Determining vaccination status and vaccine effectiveness calculation

In the pooled analysis, patients meeting the European Union ILI case definition [24] and swabbed within 7 days of symptom onset were included. Vaccination status for current (including date of vaccination and type of vaccine used) and previous season was documented either through patients' self‐report or extracted from practitioners' vaccine registries. A patient was considered vaccinated if they received at least one dose of influenza vaccine more than 14 days before symptom onset, or excluded from the analysis if vaccinated any other time in the current season. We used logistic regression to measure VE as (1 − OR) × 100. We carried out a complete case analysis. We included study site as a fixed effect and adjusted by age, sex, symptom onset time and presence of chronic condition. The functional form (categories, continuous variable or restricted cubic spline) of age and symptom onset time was determined using the Akaike information criterion.

### Prior vaccination

To study the effect of prior (2017/18) vaccination on the 2018/19 VE, we conducted an indicator analysis using four categories: individuals unvaccinated in both seasons (reference category), vaccinated in 2018/19 only, vaccinated in 2017/18 only and vaccinated in both seasons. We did not measure effect of prior (2017/18) vaccination among children younger than 9 years for whom the number of doses recommended depends on whether they were vaccinated in the previous season or not.

### Genetic characterisation and clade-specific vaccine effectiveness

Eight study sites either randomly selected specimens for sequencing or attempted to sequence the HA gene segment of all specimens. HA consensus sequences were uploaded by each site to the Global Initiative on Sharing All Influenza Data (GISAID) for ease of sharing and downloaded for centralised phylogenetic and amino acid substitution analysis of the HA1 coding portion in MEGA6 to determine clade distribution at the National Influenza Centre, Madrid. 

For the clade-specific VE analyses, we restricted the study period to weeks of symptom onset from first to last clade-specific influenza case by study site.

### Age group- and birth cohort-specific vaccine effectiveness

We measured VE by standard I-MOVE age groups (0–14, 15–64 and ≥ 65 years). To assess potential birth cohort effects, we further stratified the 15–64 years age group into three sub-categories, for which the corresponding range of birth years was derived assuming a birth year before season start (i.e. 2018 − age in years): 1987–2003, 1964–86 and 1954–63, corresponding to 15–31, 32–54 and 55–64-year-olds. The 1964–86 birth cohort (32–54-year-olds) was an adaptation of the I-REV hypothesis of first childhood imprinting with S159-bearing viruses during the ca 20-year period following the 1968 influenza A(H3N2) pandemic and is based on the analysis of historical virological sequence data (not shown) downloaded from GISAID. We gratefully acknowledge the authors, originating and submitting laboratories of these sequences from GISAID’s EpiFlu database [17]. We allowed a period of several years from birth to first influenza infection, including individuals born 4 years before the 1968 influenza A(H3N2) pandemic among those likely to be imprinted with S159-bearing viruses (i.e. those born in 1964 or later). We included in this birth cohort individuals born until and including 1986, after which there was negligible (< 5%) circulation of S159-bearing viruses. We compared age group- and birth cohort-specific VE by using an interaction between vaccination and age group/birth cohort.

### Other statistical methods

To avoid overfitting the logistic regression model, we did not attempt to measure VE if there were fewer than 10 cases or controls per number of parameters within the study site variable (N−1) in the logistic regression model. If there were fewer than 10 cases or controls per number of all parameters, we carried out a sensitivity analysis using Firth’s method of penalised regression.

### Ethical statement

The planning conduct and reporting of the studies was in line with the Declaration of Helsinki [25]. Official ethical approval was not needed in the Netherlands and Spain, as their I-MOVE studies came under the umbrella of surveillance. Other study sites sought ethical approval for their studies from a national review board, according to country-specific regulations (Croatia: Klasa 030-02/18-01/1, Ur. broj 381-10-18-2; France: #471393; Germany: EA2/126/11; Ireland: ICGP2018.4.12; Portugal: approved January 18^th^ 2012 by the Ethics Committee of Instituto Nacional de Saúde Doutor Ricardo Jorge (no registration number given); Romania: CE312/18.12.2018; Sweden: 2006/1040-31/2).

## Results

**Figure 1:**
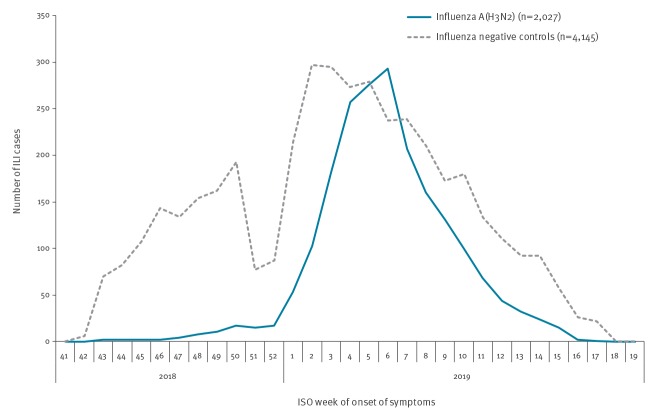
Number of ILI patients by influenza A(H3N2) status (test-negative controls and A(H3N2) cases) and week of symptom onset, I-MOVE primary care multicentre study, Europe, influenza season 2018/19 (n = 6,172)

ILI: influenza-like illness; ISO: International Organization for Standardization.

In the 2018/19 season, we included 2,027 influenza A(H3N2)-positive cases and 4,145 influenza-negative controls between ISO weeks 43/2018 and 17/2019 (Figure 1). The median age among cases was 30 years (interquartile range (IQR): 10–53 years) and the median age among controls was 31 years (IQR: 6–52 years) (Supplement 1). Among controls, 12% (490/3,931) were vaccinated compared with 14% (268/1,938) among cases. Among the 490 vaccinated controls, the influenza vaccine brand was unknown for 30%. Among the 341 with known brand, 61% (206/341) were vaccinated with a quadrivalent inactivated vaccine, 34% (117/341) were vaccinated with a trivalent non-adjuvanted inactivated vaccine, 4% (12/341) were vaccinated with a trivalent inactivated adjuvanted vaccine and 2% (6/341) were vaccinated with a live attenuated influenza vaccine. All vaccines were egg-propagated.

### Virological findings

In 2018/19 we randomly selected 575 of the 2,027 A(H3N2) viruses (28%) for sequencing (Table 1). Among those sequenced, 346 (60%) belonged to the 3C.2a1b clade and 204 (35%) belonged to the 3C.3a clade. The proportion of 3C.3a viruses was higher among 0–14-year-olds than in other age groups (56% vs 27%/24%, both p < 0.001). Among 3C.2a1b viruses, 137/346 (40%) harboured a T131K substitution and 209/346 (60%) a T135K substitution within antigenic site A in the influenza HA. 

**Table 1:** Genetic group distribution among eight study sites participating in the random sequencing of influenza virus-positive specimens overall and by age group, I-MOVE primary care multicentre study, Europe, influenza season 2018/19 (n = 575)

Characterised viruses^a^	Clade/subclade	All ages		0–14 years		15–64 years		≥65 years	
		n	%	n	%	n	%	n	%
A/Alsace/1746/2018	3C.2a1b	346	60	71	40	214	68	61	73
*+ T131K* ^b^		*137*	*40*	*34*	*48*	*83*	*39*	*20*	*33*
*+ T135K* ^b^		*209*	*60*	*37*	*52*	*131*	*61*	*41*	*67*
A/Switzerland/8060/2017	3C.2a2	8	1	1	1	6	2	1	1
A/Cote d'Ivoire/544/2016	3C.2a3	16	3	5	3	10	3	1	1
A/Valladolid/182/2017	3C.2a4	1	0	1	1	0	0	0	0
A/England/538/2018	3C.3a	204	35	98	56	86	27	20	24
All sequenced		575	100	176	100	316	100	83	100

**^a^** All 3C.2a1b, 3C.2a2, 3C.2a3, 3C.2a4 viruses bear Y159 in antigenic site B in the influenza haemagglutinin (HA). Of the 204 3C.3a viruses, 203 were S159-bearing, one 3C.3a virus was F159-bearing. Of the 209 3C.2a1b + T135K viruses, 196 also harboured the T128A substitution in antigenic site A in the HA.

^b^ Positions 131 and 135 are within antigenic site A in the HA. The rows entitled +T131K and +T135K contain counts of genetic variants of A/Alsace/1746/2018 and together, their counts equal the total in the row of A/Alsace/1746/2018.

In the complete case analysis we dropped 5% (110/2,027) of cases and 6% (260/4,145) of controls because of missing values in key covariates.

The 2018/19 end-of-season VE against influenza A(H3N2) among all ages was −1% (95% CI: −24 to 18) (Table 2). VE among 15–64 year olds was −26% (95% CI: −66 to 4), lower than the VE among 0–14-year-olds (46%; 95% CI: 8–68; p value for difference to 15–64-year-olds: p = 0.020) and among those 65 years and older (20%; 95% CI: −20 to 46; p value for difference to 15–64-year-olds: p = 0.321) (Table 2).

**Table 2:** Pooled adjusted seasonal vaccine effectiveness against influenza A(H3N2), overall, by age groups, by clade and genetic variants, I-MOVE primary care multicentre study, Europe, influenza season 2018/19 (n = 5,802)

**Age group**	**Outcome**	n	**Cases**		**Controls**		**Adjusted VE (%)**	**95% CI (%)**
			**All**	**Vaccinated**	**All**	**Vaccinated**		
All ages	A(H3N2)	5,802	1,917	265	3,885	485	−1	−24 to 18
0–14 years		2,008	668	33	1,340	62	46	8–68
15–64 years		3,153	1,038	123	2,115	194	−26	−66 to 4
≥ 65 years		641	211	109	430	229	20	−20 to 46
All ages	A(H3N2) clade 3C.2a1b	3,217	334	55	2,883	375	28	−7 to 51
15–64 years		1,799	211	26	1,588	159	15	−43 to 50
All ages	A(H3N2) clade 3C.2a1b + T131K^a^	2,582	131	15	2,451	329	57	16–78
15–64 years		1,468	81	7	1,387	141	51	−21 to 80
All ages	A(H3N2) clade 3C.2a1b + T135K^a^	2,764	203	40	2,561	342	7	−52 to 43
15–64 years		1,515	130	19	1,385	145	−7	−102 to 43
All ages	A(H3N2) clade 3C.3a	2,000	201	35	1,799	270	−42	−137 to 15
0–14 years		715	97	8	618	41	42	−58 to 78
15–64 years		1,082	84	12	998	115	−74	−259 to 6

^a^ Positions 131 and 135 are in antigenic site A in the influenza haemagglutinin.

CI: confidence interval; VE: vaccine effectiveness.

**Figure 2:**
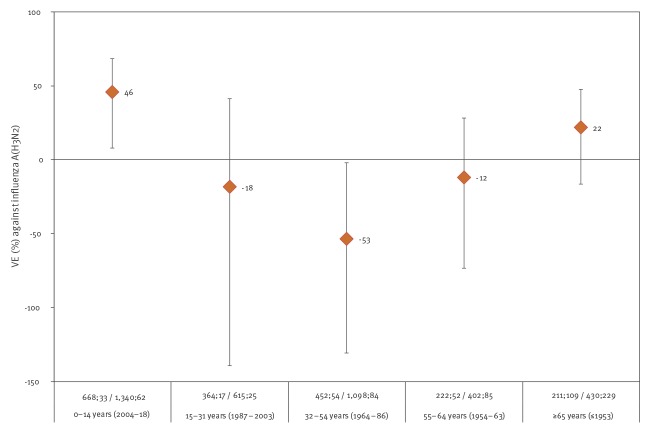
Birth cohort-specific vaccine effectiveness against influenza A(H3N2), I-MOVE primary care multicentre study, Europe, influenza season 2018/19 (n = 5,802)

Among the birth cohort 1964–86 (32–54-year-olds), i.e. those potentially exposed in childhood to an S159-bearing A(H3N2) virus, the VE was −53% (95% CI: −131 to −2) (Figure 2). The VE among those born 1987–2003 (15–31-year-olds) and 1954–63 (55–64-year-olds) was −18% (95% CI: −140 to 41) and −12% (95% CI: −74 to 28), respectively. The same pattern is seen when modelling influenza A(H3N2) VE by year of age; further details are listed in Supplement 2.

VE: vaccine effectiveness.

Numbers on the x axis represent: cases; vaccinated cases / controls; vaccinated controls.

### Clade-specific vaccine effectiveness

VE against clade 3C.2a1b was 28% (95% CI: −7 to 51) among all ages, with a VE of 15% (95% CI: −43 to 50) among those aged 15–64 years (Table 2). VE against clade 3C.2a1b with the T131K substitution among all ages was 57% (95% CI: 16–78) and among those aged 15–64 years 51% (95% CI: −21 to 80). The VE against clade 3C.2a1b with the T135K substitution was 7% (95% CI: −52 to 43) among all ages and among those aged 15–64 years −7% (95% CI: −102 to 43).

VE against clade 3C.3a was −42% (95% CI: −137 to 15) among all ages, −74% (95% CI: −259 to 16) among those aged 15–64 years, and 42% (95% CI: −58 to 78) among those aged 0–14 years. Because of the small sample size, further age stratification was not carried out.

### Vaccine effectiveness against influenza A(H3N2) and subclades by previous vaccination status

The VE against clade 3C.2a1b among those vaccinated in the 2018/19 season only was −14% (95% CI: −132 to 44) and 49% (95% CI: 17–69) among those vaccinated in the 2017/18 and 2018/19 seasons (Table 3).

**Table 3:** Pooled adjusted seasonal vaccine effectiveness against influenza A(H3N2), by previous vaccination status, among those aged ≥ 9 years and by age group, by clade and genetic variants, I-MOVE primary care multicentre study, Europe, influenza season 2018/19 (n = 3,983)

Age group	Outcome	Previous vaccination status	Cases	Controls	Adjusted VE (%)	95% CI (%)
≥ 9 years	A(H3N2)	Not vaccinated in either season	1,147	2,061	Ref	
		Vaccinated in 2018/19 only	76	99	−27	−81 to 11
		Vaccinated in 2017/18 only	25	113	45	11 to 66
		Vaccinated in both seasons	153	309	5	−23 to 27
15–64 years	A(H3N2)	Not vaccinated in either season	852	1,719	Ref	
		Vaccinated in 2018/19 only	46	59	−43	−121 to 7
		Vaccinated in 2017/18 only	17	79	46	5 to 70
		Vaccinated in both seasons	69	116	−14	−62 to 20
≥ 65 years	A(H3N2)	Not vaccinated in either season	91	152	Ref	
		Vaccinated in 2018/19 only	24	36	22	−62 to 63
		Vaccinated in 2017/18 only	5	30	48	−55 to 83
		Vaccinated in both seasons	78	184	31	−9 to 56
≥ 9 years	Clade 3C.2a1b	Not vaccinated in either season	209	1,492	Ref	
		Vaccinated in 2018/19 only	12	80	−14	−132 to 44
		Vaccinated in 2017/18 only	4	74	ND^a^	
		Vaccinated in both seasons	36	203	49	17 to 69
≥ 9 years	Clade 3C.3a	Not vaccinated in either season	94	930	Ref	
		Vaccinated in 2018/19 only	6	58	−34	−248 to 49
		Vaccinated in 2017/18 only	3	42	ND^a^	
		Vaccinated in both seasons	20	163	−122	−353 to −9

^a^ Omitted because of small sample size.

CI: confidence interval; ND: not done; Ref: reference value; VE: vaccine effectiveness.

The VE against clade 3C.3a among those vaccinated in the 2018/19 season only was −34% (95% CI: −248 to 49) and −122% (95% CI: −353 to −9) among those vaccinated in the 2017/18 and 2018/19 seasons.

## Discussion

The VE against influenza A(H3N2) in the 2018/19 I-MOVE study was low overall. Results suggest that this was driven by the age group 15–64 years, in whom the 2018/19 influenza vaccine was not effective against influenza A(H3N2). The lack of VE against influenza A(H3N2) among 15–64-year-olds has not been seen before in I-MOVE studies [26-29]. The VE against influenza A(H3N2) in this age group in 2016/17 and 2017/18, when the A/Hong Kong/4801/2014 (H3N2)-like vaccine virus component was used (a 3C.2a clade virus) and predominantly 3C.2a (sub)clades circulated, was 34% and 33%, respectively [5]. The 2018/19 VE was higher in flanking age groups. This pattern was also observed in Canada in VE against influenza A(H3N2), the US outpatient VE study against influenza A(H3N2)and the US inpatient VE study against any influenza [7,8,30]. In the latter study, the VE against influenza A(H3N2) was −43% (95% CI: −102 to −2).

An overall low VE was expected because of egg propagation of the vaccine seed virus and the co-circulation of clade 3C.3a viruses that are genetically distinct from the vaccine virus clade [2]. In addition, even among the 3C.2a1b viruses, which are genetically closer to the vaccine virus, two distinct clusters have emerged and point estimates indicate that the T135K-harbouring genetic variant may have a lower VE than the T131K-harbouring genetic variant. The T135K-harbouring genetic variant most often also harboured the T128A substitution; both mutations result in loss of glycosylation sequons [31], which can be associated with antigenic change. But these factors alone are not sufficient to explain the observed age-specific difference in VE against influenza A(H3N2) in 2018/19.

In this 2018/19 season, VE against clade 3C.3a was very low among 15–64-year-olds, indicating that clade 3C.3a viruses played a major role in the observed VE against any influenza A(H3N2) in this age group. The marked differences in point estimates among 0–14- and 15–64-year-olds in VE against clade 3C.3a may indicate a birth cohort effect. When stratifying the age group 15–64 years according to birth cohorts who had or had not been potentially imprinted with A(H3N2) viruses bearing S159 in antigenic site B in the HA, we observe the lowest VE point estimate among those born 1964–86 (32–54-year-olds; those with the potential exposure to S159-bearing A(H3N2) virus). This dip in VE is consistent with the I-REV hypothesis [7]. The low VE against influenza A(H3N2) among those born 1954–63 (aged 55–64 years) could be explained in part by random variation because of low sample size. Also, these individuals are likely to have been imprinted in early childhood by A(H2N2), followed by potential re-infection with a S159-bearing A(H3N2) influenza virus. The analogous position to 159 in antigenic site B in the A(H2N2) HA is 154. The predominant amino acid at this position was S during the influenza A(H2N2) pandemic in 1957/58 and during early influenza A(H2N2) circulation, moving to proline (P) and glutamine (Q) in later years; this position is associated with antigenic cluster transition [32,33]. It is unclear if imprinting with S154-bearing A(H2N2) viruses may have had a similar effect to imprinting with S159-bearing A(H3N2) viruses as so few patients in the study constitute the 1954–63 birth cohort and among those, only a subset would have been infected with S154-bearing A(H2N2) viruses. In addition, it has been suggested that infection with pandemic influenza A(H2N2) after likely first imprinting with influenza A(H1N1) may give rise to long-lasting immunological influenza A(H2N2) imprinting [33]. Again, we are unable to determine if the low VE among the 1954–63 birth cohort was analogously due to immunological imprinting by S159-bearing A(H3N2) viruses after first imprinting with A(H2N2) viruses, however further research in this field may be warranted.

If infection by S159-bearing A(H3N2) viruses were the only factor influencing the observed age-specific differences in VE, then we would expect a higher VE among those born 1987–2003 (aged 15–31 years), who were not likely to be imprinted by S159-bearing A(H3N2) viruses in childhood. While the VE among those born 1987–2003 (aged 15–31 years) was higher than that among those born 1964–86 (aged 32–54 years), it was still low and not statistically significantly different. The lack of difference in estimates could be in part explained by a dilution effect of both 3C.3a and 3C.2a1b co-circulating among adult age groups during the 2018/19 season and in particular also by sample size issues relating to the low vaccination coverage in this group in our study. Notably, as suggested by Skowronski et al. [7], the influence of substitutions at positions other than 159, such as 193, also within the antigenic site B in the HA, may further explain the low VE among those born 1987–2003 (aged 15–31 years). The pattern of VE by year of age as illustrated in Supplementary Figure S2A, is what we would expect to see if there was a strong effect of S159-bearing viruses among those born in 1964–86 and also an effect of S193-bearing viruses in 1991–2005. The gentle slope upwards among those born in 1954–63 is also compatible with the plausible assumption that some individuals in this birth cohort potentially had their first influenza infection (and were imprinted) by an A(H3N2) S159-bearing influenza virus after the age of 5 years and thus drive the VE estimates downwards. This analysis of VE by year of age is, however, sensitive to knot position (as outlined in Supplement 2) and should be interpreted with caution.

While being vaccinated in the 2017/18 season improved 2018/19 VE against clade 3C.2a1b, it had the opposite effect against clade 3C.3a. Repeat vaccination, with the 2017/18 and 2018/19 Y159-containing vaccines, in addition to childhood imprinting, may be a further piece of the puzzle explaining the low VE observed against influenza A(H3N2) in 2018/19, as also suggested by Skowronski et al. as a potential immunological mechanism underpinning their I-REV hypothesis [7].

The low VE among 15–64-year-olds may also be explained by bias. In I-MOVE countries, this age group is not part of a universal influenza vaccination recommendation. In this age group, only people with pre-existing medical conditions (e.g. cardiorespiratory diseases, pregnancy) and healthcare workers are recommended and offered vaccinations in these countries [34]. The VE in the target group for vaccination among the 15–64 year age group was −5% (see Supplement 3) and while still low, it was higher than the VE among 15–64-year-olds in the general population. However, in this age group, the target group for vaccination had a lower percentage of clade 3C.3a-infected persons (20% vs 30%). Importantly, the small sample size limits the inferences we can draw about birth cohort effects.

More research needs to be carried out into selection bias and sparse data bias. We attempted to assess sparse data bias, by comparing standard logistic regression to penalised logistic regression whenever models may have been overfitted. While the VE of the penalised logistic regression was always more towards the null, the absolute difference between the two VE estimates was always < 4%. The I-MOVE study is subject to the usual biases of observational studies, however it is a well-established and stable study. Age-specific VE differences to the extent observed for influenza A(H3N2) in 2018/19 were not observed for influenza A(H1N1)pdm09 in the same season, nor for influenza A(H3N2) in any previous season, which makes it less likely that bias was a major explanatory factor. Small sample size limits the precision of our birth cohort-specific estimates and some confidence limits overlap. Despite this, we still observe a significant difference in VE not only between the youngest and middle age group (0–14 years vs 15–64 years; p = 0.020), but also between the 2004–18 (0–14-year-olds) and 1964–86 (32–54-year-olds) birth cohorts (p value for difference = 0.036), as point estimates are so different. Overlapping confidence intervals among birth cohorts within the middle age group are expected given the small sample size and the expectation of low VE among several birth cohorts (those potentially infected with S159-bearing and those potentially infected with S193-bearing viruses) according to the I-REV hypothesis.

Further investigations are necessary to understand these age-specific differences in VE against influenza A(H3N2) in the 2018/19 season, including further analysis of the effects of previous vaccination and a more in-depth analysis of birth cohorts and virological changes in Europe over time. These analyses could include a focus on amino acid changes at key positions, but also the influence of influenza A(H1N1), A(H2N2) and B virus imprinting on the 2018/19 VE against influenza A(H3N2) [18,35]. To better understand birth cohort effects in influenza, we welcome the two US National Institutes of Health-funded studies among infants [36]; however, we would also welcome a European longitudinal study among working age adults, to better understand how influenza imprinting and prior and repeated vaccination may affect future VE when this cohort becomes part of the target group for vaccination. This study also highlights the importance of sequencing viruses included in a VE study as this can be crucial to interpretation of VE findings and variation by sub-strata.

Our results suggest that influenza A(H3N2) VE may be low among middle-aged adults in subsequent seasons if a Y159-bearing vaccine virus (e.g. 3C.2a1 subclade) is used and 3C.3a or S159-bearing viruses are circulating. The reverse scenario (e.g. 3C.3a vaccine and circulating 3C.2a1 viruses, potentially applicable to the 2019/20 season), merits consideration, as the influenza A(H3N2) VE may also be low. In these two scenarios and if the season is dominated by influenza A(H3N2), early recommendations for other prophylactic measures, such as use of neuraminidase inhibitors as prophylaxis or treatment among risk populations regardless of vaccination status, should be considered, even among younger adults. We encourage other study sites to measure birth cohort-specific VE in the 2019/20 and subsequent seasons to try and understand more about mechanisms of birth cohort-specific effects.

## I-MOVE primary care study team

Germany

Ute Preuss, Department for Infectious Disease Epidemiology, Respiratory Infections Unit, Robert Koch Institute, Germany

Kristin Tolksdorf, Department for Infectious Disease Epidemiology, Respiratory Infections Unit, Robert Koch Institute, Germany

Barbara Biere, National Reference Center for Influenza, Robert Koch Institute, Germany

Maria Smallfield, National Reference Center for Influenza, Robert Koch Institute, Germany

Marianne Wedde, National Reference Center for Influenza, Robert Koch Institute, Germany

France

Alessandra Falchi, Laboratoire de Virologie, Université de Corse-Inserm, Corte, France

Shirley Masse, Laboratoire de Virologie, Université de Corse-Inserm, Corte, France

Natacha Villechenaud, Laboratoire de Virologie, Université de Corse-Inserm, Corte, France

Cécile Souty, Sorbonne Université, INSERM, Institut Pierre Louis d’Epidémiologie et de Santé Publique (IPLESP), F75012 Paris, France

Thierry Blanchon, Sorbonne Université, INSERM, Institut Pierre Louis d’Epidémiologie et de Santé Publique (IPLESP), F75012 Paris, France

Titouan Launay, Sorbonne Université, INSERM, Institut Pierre Louis d’Epidémiologie et de Santé Publique (IPLESP), F75012 Paris, France

Vincent Enouf, Unité de Génétique Moléculaire des Virus à ARN, UMR 3569 CNRS, Université Paris Diderot SPC, Institut Pasteur, Paris, France; CNR des virus des infections respiratoires, Institut Pasteur, Paris, France

Sylvie Behillil, Unité de Génétique Moléculaire des Virus à ARN, UMR 3569 CNRS, Université Paris Diderot SPC, Institut Pasteur, Paris, France; CNR des virus des infections respiratoires, Institut Pasteur, Paris, France

Bruno Lina, Laboratoire de Virologie, CNR des virus des infections respiratoires, Institut des Agents Infectieux, Groupement Hospitalier Nord des HCL, Lyon, France; Laboratoire Virpath, CIRI Inserm U1111, CNRS 5308, ENS, UCBL, Faculté de Médecine LYON Est, Université de Lyon, Lyon, France

Martine Valette, Laboratoire de Virologie, CNR des virus des infections respiratoires, Institut des Agents Infectieux, Groupement Hospitalier Nord des HCL, Lyon, France

Spain

Clara Mazagatos, National Centre of Epidemiology, Institute of Health Carlos III, CIBERESP, Spain

Inmaculada Casas, National Centre for Microbiology, National Influenza Reference Laboratory, WHO-National Influenza Centre, Institute of Health Carlos III, Spain

Luis García Comas, Dirección General de Salud Pública, Comunidad de Madrid, Spain

María Esther Insua Marisquerena, Dirección General de Salud Pública, Comunidad de Madrid, Spain

Juan Carlos Galán, Laboratorio Hospital Ramón y Cajal, CIBERESP, Spain

Jesús Castilla, Instituto de Salud Pública de Navarra - IdiSNA, and CIBERESP, Pamplona, Spain

Manuel García Cenoz, Instituto de Salud Pública de Navarra - IdiSNA, and CIBERESP, Pamplona, Spain Ana Navascués, Complejo Hospitalario de Navarra – IdiSNA, Pamplona, Spain

Carmen Quiñones Rubio, Dirección General de Salud Pública y Consumo de La Rioja, Spain

Ana Carmen Ibañez Perez, Dirección General de Salud Pública y Consumo de La Rioja, Spain

Eva Martinez Ochoa, Dirección General de Salud Pública y Consumo de La Rioja, Spain

Miriam Blasco, Laboratorio Hospital San Pedro de Logroño, Spain

Jaume Giménez Duran, Servicio de Epidemiología, Dirección General de Salud Pública, Mallorca, Spain

Juana Maria Vanrell, Servicio de Epidemiología, Dirección General de Salud Pública, Mallorca, Spain

Jordi Reina, Laboratorio del Hospital Son Espases, Mallorca, Spain

Daniel Castrillejo, Servicio de Epidemiología. DGSC, Consejería de Bienestar Social y Sanidad, Ciudad Autónoma de Melilla, Spain

Sweden

Åsa Wiman, Public Health Agency of Sweden, Stockholm, Sweden

Ireland

Meadhbh Hunt, HSE-Health Protection Surveillance Centre, Ireland

Michael Joyce, Irish College of General Practitioners, Ireland

Olga Levis, Irish College of General Practitioners, Ireland

Claire Collins, Irish College of General Practitioners, Ireland

Linda Dunford, National Virus Reference Laboratory, University College Dublin, Ireland

Charlene Bennett, National Virus Reference Laboratory, University College Dublin, Ireland

Joanne Moran, National Virus Reference Laboratory, University College Dublin, Ireland

Grainne Tuite, National Virus Reference Laboratory, University College Dublin, Ireland

Jeff Connell, National Virus Reference Laboratory, University College Dublin, Ireland

Cillian de Gascun, National Virus Reference Laboratory, University College Dublin, Ireland

Portugal

Ana Paula Rodrigues, Departamento de Epidemiologia, Instituto Nacional de Saúde Dr Ricardo Jorge, Portugal

Ausenda Machado, Departamento de Epidemiologia, Instituto Nacional de Saúde Dr Ricardo Jorge, Portugal

Baltazar Nunes, Departamento de Epidemiologia, Instituto Nacional de Saúde Dr Ricardo Jorge, Portugal

Irina Kislaya, Departamento de Epidemiologia, Instituto Nacional de Saúde Dr Ricardo Jorge, Portugal

Inês Costa, Departamento de Doenças Infeciosas, Instituto Nacional de Saúde Dr Ricardo Jorge, Portugal

Patrícia Conde, Departamento de Doenças Infeciosas, Instituto Nacional de Saúde Dr Ricardo Jorge, Portugal

Paula Cristóvão, Departamento de Doenças Infeciosas, Instituto Nacional de Saúde Dr Ricardo Jorge, Portugal

Pedro Pechirra, Departamento de Doenças Infeciosas, Instituto Nacional de Saúde Dr Ricardo Jorge, Portugal

Vítor Borges, Departament of Infectious Diseases/Bioinformatics Unit, Instituto Nacional de Saúde Dr Ricardo Jorge, Portugal

The Netherlands

Mariam Bagheri, National Institute for Public Health and the Environment (RIVM), Bilthoven, the Netherlands

Sharon van den Brink, National Institute for Public Health and the Environment (RIVM), Bilthoven, the Netherlands

Frederika Dijkstra, National Institute for Public Health and the Environment (RIVM), Bilthoven, the Netherlands

Gabriel Goderski, National Institute for Public Health and the Environment (RIVM), Bilthoven, the Netherlands

Wim van der Hoek, National Institute for Public Health and the Environment (RIVM), Bilthoven, the Netherlands

Marit de Lange, National Institute for Public Health and the Environment (RIVM), Bilthoven, the Netherlands

Ton Marzec, National Institute for Public Health and the Environment (RIVM), Bilthoven, the Netherlands

Pieter Overduin, National Institute for Public Health and the Environment (RIVM), Bilthoven, the Netherlands

Daphne Reukers, National Institute for Public Health and the Environment (RIVM), Bilthoven, the Netherlands

Anne Teirlinck, National Institute for Public Health and the Environment (RIVM), Bilthoven, the Netherlands

Lisa Wijsman, National Institute for Public Health and the Environment (RIVM), Bilthoven, the Netherlands

Gé Donker, Nivel (the Netherlands Institute for Health Services Research), Utrecht, the Netherlands

Romania

Maria Elena Mihai, “Cantacuzino” National Military-Medical Institute for Research and Development, Bucharest, Romania

Carmen Maria Cherciu, “Cantacuzino” National Military-Medical Institute for Research and Development, Bucharest, Romania

Viorel Alexandrescu, “Cantacuzino” National Military-Medical Institute for Research and Development, Bucharest, Romania

Croatia

Bernard Kaic, Croatian Institute of Public Health, Division for epidemiology of communicable diseases, Zagreb, Croatia

Sanja Kurecic Filipovic, Croatian Institute of Public Health, Division for epidemiology of communicable diseases, Zagreb, Croatia

Iva Pem Novosel, Croatian Institute of Public Health, Division for epidemiology of communicable diseases, Zagreb, Croatia

Zvjezdana Lovric Makaric, Croatian Institute of Public Health, Division for epidemiology of communicable diseases, Zagreb, Croatia

Martina Zajec, Croatian Institute of Public Health, Division for epidemiology of communicable diseases, Zagreb, Croatia

Vladimir Draženović, Croatian Institute of Public Health, Virology Department, Zagreb, Croatia

Epiconcept

Alain Moren, Epiconcept, Paris, France

## Supplementary Data

481000 Supplements_Kissling
