# Supplementary material for: Low 2018/19 vaccine effectiveness against influenza A(H3N2) among 15–64-year-olds in Europe: exploration by birth cohort
Source: Euro Surveill. 2019 Nov 28;24(48):1900604. doi: 10.2807/1560-7917.ES.2019.24.48.1900604 (PMC6891946; doi:10.2807/1560-7917.ES.2019.24.48.1900604)
Supplement: Supplements_Kissling [file 19-00604_KISSLING_Supplements.pdf]

Supplementary material for the article “**Low 2018–19 influenza vaccine effectiveness against A(H3N2) among 15–64-year-olds in Europe: exploration by birth cohort**“

This supplementary material is hosted by *Eurosurveillance* as supporting information alongside the article “**Low 2018–19 influenza vaccine effectiveness against A(H3N2) among 15–64-year-olds in Europe: exploration by birth cohort**“, on behalf of the authors, who remain responsible for the accuracy and appropriateness of the content. The same standards for ethics, copyright, attributions and permissions as for the article apply. Supplements are not edited by *Eurosurveillance* and the journal is not responsible for the maintenance of any links or email addresses provided therein.

Supplement 1: Participant profile among A(H3N2) cases and test-negative controls, I-MOVE primary care multicentre study, Europe, influenza season 2018–19

**Variables**	Number of influenza A(H3N2) cases /total		Number of test-negative controls /total	
	N	%	N	%
**Age groups**				
0-4	270/2,027	13	939/4,134	23
5-14	425/2,027	21	486/4,134	12
15-64	1,087/2,027	54	2,226/4,134	54
≥ 65	245/2,027	12	483/4,134	12
Missing	0	-	11	-
**Sex**				
Female	1,076/2,022	53	2,237/4,131	54
Missing	5	-	14	-
**Days between onset of symptoms and swabbing**				
0	101/2,027	5	209/4,145	5
1	737/2,027	36	1,336/4,145	32
2	622/2,027	31	1,090/4,145	26
3	330/2,027	16	687/4,145	17
4-7	237/2,027	12	823/4,145	20
**Seasonal vaccination, 2018–19**	268/1,938	14	490/3,931	12
Missing	89	-	214	-
**Seasonal vaccination, 2017–18**	232/1,907	12	532/3,847	14
Missing	120	-	298	-
**Seasonal vaccination group among vaccinated**				

**Variables**	Number of influenza A(H3N2) cases /total		Number of test-negative controls /total	
	N	%	N	%
Trivalent subunit	47/267	17	70/490	14
Trivalent split virion	39/267	15	47/490	10
Trivalent adjuvanted	2/267	1	12/490	2
Quadrivalent subunit	30/267	11	86/490	18
Quadrivalent split virion	56/267	21	120/490	25
LAIV	0/267	0	6/490	1
Unknown	94/267	35	149/490	30
Missing vaccination date or status	89	-	214	-
**At least one chronic condition**	387/2,007	19	837/4,102	20
Missing	20	-	43	-
**At least one hospitalisation in the previous 12 months for chronic conditions**	20/1,917	1	57/4,097	1
Missing	110	-	197	-
**Belongs to target group for vaccination**	546/2,021	27	1,144/4,108	28
Missing	6	-	37	-

Supplement 2: VE against A(H3N2) by year of age, I-MOVE primary care multicenter study, Europe, influenza season 2018–19

To model VE against A(H3N2) continuously by year of age, we created a restricted cubic spline of age and modelled the interaction between current seasonal influenza vaccination and the age spline. We chose the position of knots according to Harrell's centiles [2] to ensure best data fit, and added an extra knot in the middle of the 32–54 year old cohort, to ensure an adequate model by birth cohort. In addition we ensured that none of the standard errors in the spline coefficients exceeded the coefficient parameter (a sign of overfitting).

Our best and alternative models are presented in Figures S3A–C, along with the AIC and position of knots.

Figure S2A: VE against A(H3N2) by year of birth, using a spline with 6 knots, I-MOVE primary care multicenter study, Europe, influenza season 2018–19 (model of choice)

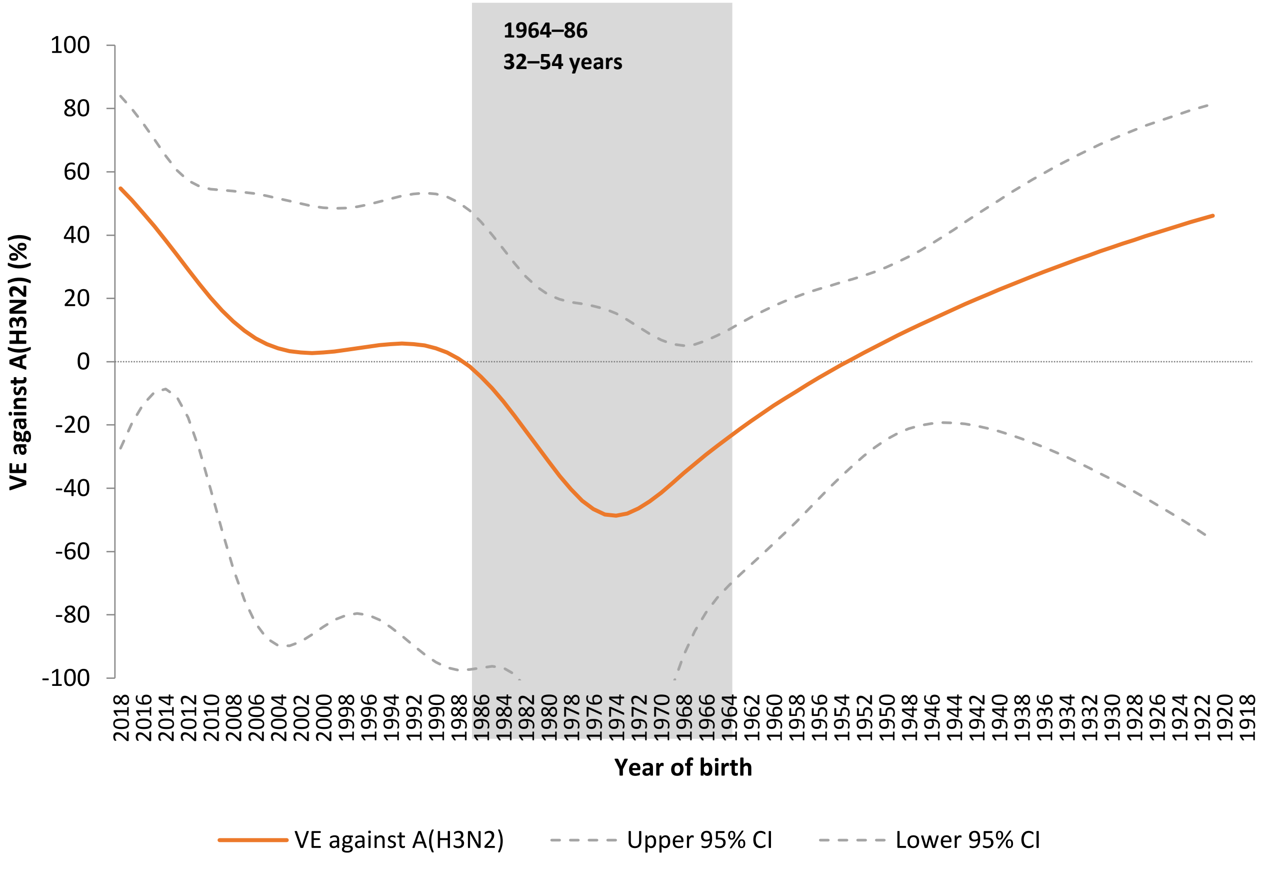

Knot positions: 1 9 30 43 50 73
AIC: 6041

Figure S2B: VE against A(H3N2) by year of birth, using a spline with 5 knots, I-MOVE primary care multicenter study, Europe, influenza season 2018–19

The position of the knots were defined by Harrell's percentiles.

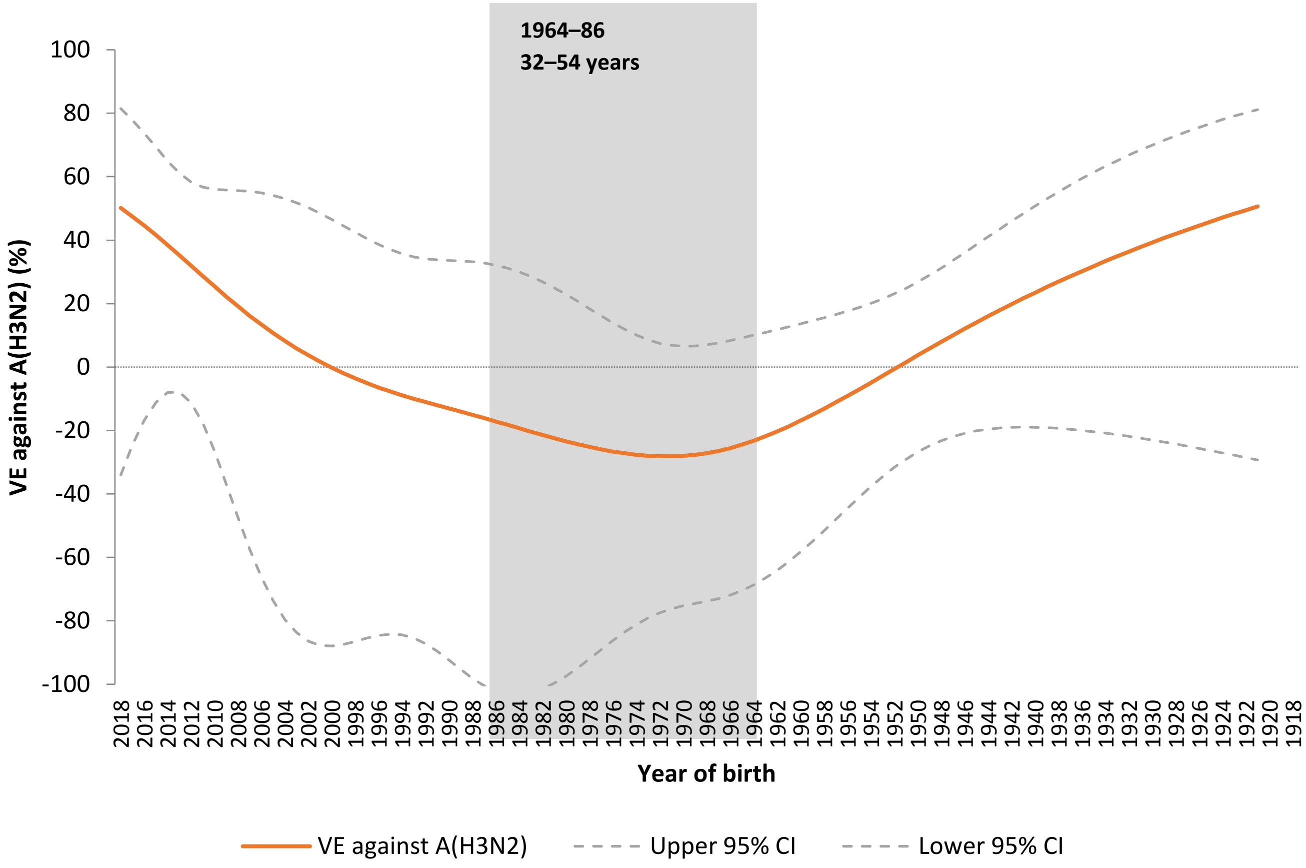

Knot positions: 1 9 30 50 73
AIC: 6037

Figure S2C: VE against A(H3N2) by year of birth, using a spline with 4 knots, I-MOVE primary care multicenter study, Europe, influenza season 2018–19

The position of the knots were defined by Harrell's percentiles.

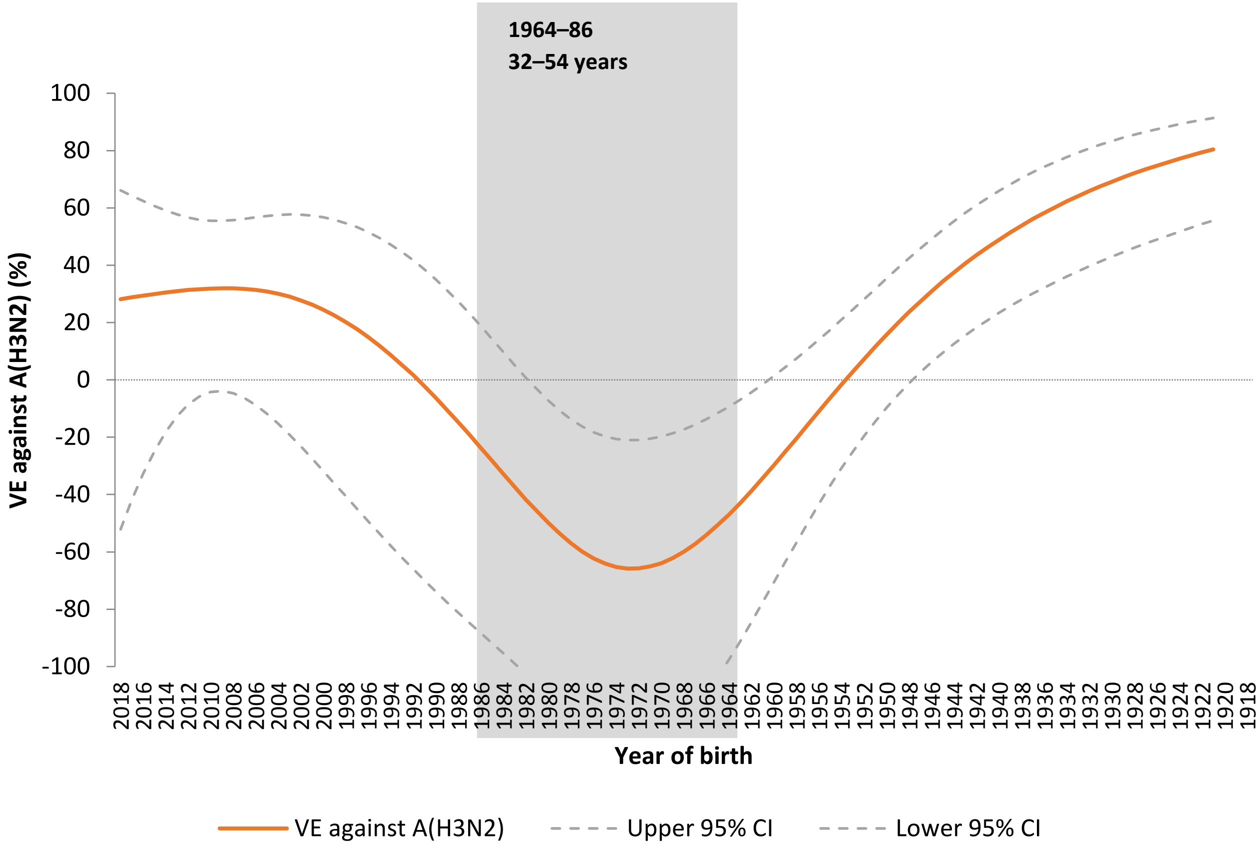

Knot positions: 1 15 43 73
AIC: 6102

Supplement 3: VE against A(H3N2) among the target group for vaccination, overall and by age group, I-MOVE primary care multicenter study, Europe, influenza season 2018–19

The VE among the target group for vaccination is similar among the 0–14 age group compared to the general population (45% vs. 46%), but lower among 15–64 year olds (-5% vs. -26%). If clade 3C.3a strongly contributes to the low VE among 15–64 year olds, then the difference in VE in this age group among the target group for vaccination compared to the general population could be in part explained by the difference in distribution of 3C.3a vs. other clades. In the target group for vaccination, 24% of patients in the complete case analysis have viruses belonging to the 3C.3a clade. In the general population, 32% of patients in the complete case analysis have viruses belonging to the 3C.3a clade.

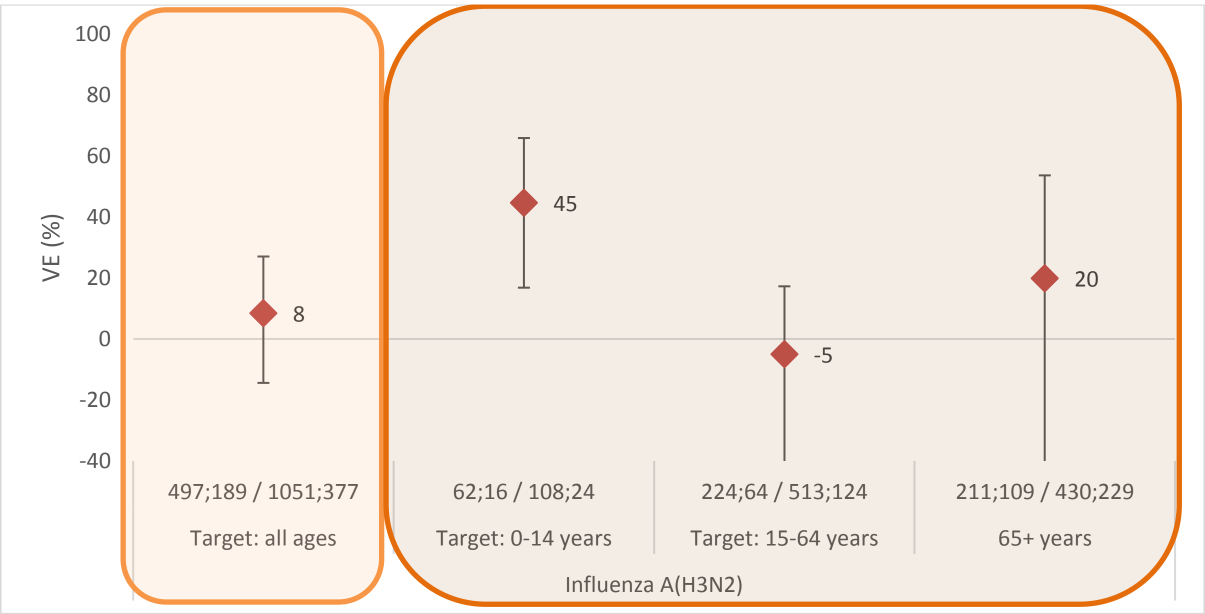

References

[1] Y. Shu and J. McCauley, ‘GISAID: Global initiative on sharing all influenza data – from vision to reality’, *Euro Surveill.*, vol. 22, no. 13, p. 30494, Mar. 2017.

[2] F. E. Harrell, *Regression modeling strategies: with applications to linear models, logistic regression, and survival analysis*. New York: Springer, 2001.
